# Combined Effects of Zein Nanofiber Coating Containing Laurel (*Laurus nobilis*) and Air Fryer Cooking on Quality Properties of Fish Fillets during Cold Storage

**DOI:** 10.1021/acsomega.3c06318

**Published:** 2024-02-14

**Authors:** Zafer Ceylan, Raciye Meral, Aslıhan Alav, Gülşen Berat Torusdağ, Fatih Bildik, Filiz Altay

**Affiliations:** † Science Faculty, Department of Molecular Biology and Genetics/Biotechnology, Bartın University, Bartın 74100, Türkiye; ‡ Faculty of Engineering, Department of Food Engineering, Van Yuzuncu Yıl University, Tuşba, Van 65080, Türkiye; § Institute of Science, Department of Food Engineering, Van Yuzuncu Yıl University, Tuşba, Van 65080, Türkiye; ∥ Faculty of Tourism, Department of Gastronomy, Van Yuzuncu Yıl University, Tuşba, Van 65080, Türkiye; ⊥ Faculty of Chemical and Metallurgical Engieering, Department of Food Engineering, Istanbul Technical University, Maslak, Sarıyer, Istanbul 34469, Turkey

## Abstract

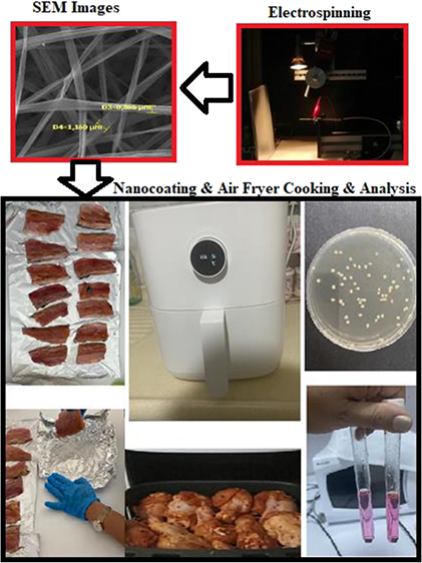

In this study, the effects of zein nanofibers (Zn) containing ground laurel leaves (GLL) and air fry cooking on the quality characteristics of Rainbow trout (*Oncorhynchus mykiss*) were investigated. The zein nanofibers possessing 335.8 ± 43.6 nm average diameters were fabricated containing GLL. The Fourier transform infrared spectroscopy (FTIR) results of the zein, Zn, GLL, and zein nanofibers containing GLL (LZn) confirmed the electrospinning encapsulation of GLL into Zn and their interactions. The effects of the combination of LZn coating and air fryer cooking of fish fillets on the quality characteristics during storage at 4 °C for 10 days were monitored in terms of oxidative and microbiological stability, color, and sensory parameters. As compared to the control, the combination of LZn coating and air fryer cooking provided a microbial limitation of up to 45.21% during the analysis (*p* < 0.05). The changes in Δ*E* values between the control and the LZn-coated samples were obtained as ≤7.56 during 6 days, but then a dramatic color difference was observed. Besides overall sensory acceptability, particularly the odor parameter in the cooked fish samples coated with LZn was significantly preferred (*p* < 0.05). The combination of LZn coating and air fryer cooking delayed the thiobarbituric acid increase in the fish meat samples (3.51 to 2.57 mg malondialdehyde (MDA)/kg) up to the third day of storage. This study showed that LZn coating is a very functional layer on the fish meat and could be applied for not only fresh fish meat but also other fresh meat products.

## 1 Introduction

*Laurus nobilis* L. (bay, bay laurel, or daphne) belongs to the Lauraceae family and is grown in East Asia and South and North America.^1^*L. nobilis* leaves have been traditionally used as a flavoring agent in Mediterranean cuisine^2^ and as an ingredient in folk medicine for the treatment of various diseases such as viral infections, cough, rheumatism, impaired digestion, and diarrhea.^3^ The antimicrobial,^4,5^ antifungal,^6,7^ anticonvulsant,^8^ antioxidant,^9,10^ anti-inflammatory,^11,12^ antidiabetic,^13−15^ anticancer,^16,17^ neuroprotective,^18^ and anticholinergic^19^ effects of *L. nobilis* have been reported in the literature.

Laurel leaves are preferred in fish courses^20,21^ due to the masking of sharp and distinct fish aroma during cooking. Laurel leaves are present when frying fish in a pan or cooker, but since they cannot be consumed directly due to their bitter flavor,^22^ they are mostly used for their aroma. There are studies about laurel leaf extract and laurel essential oils; there is no investigation on laurel leaves and their interactions with a food product in terms of their functional effects. Besides the potential use of laurel leaves in the different fish courses, corn meal is used for coating fish fillets, especially before being fried in Turkiye and Greece. This way, corn meal has a kind of protection for fish from heat treatment and also contributes to the taste, including its sandy texture on the surface of the fish. Zein is a protein that corresponds to 50% of the total protein content of corn. To illustrate heating treatment combined with nanotechnology, cooked salmon and red meat samples coated with nanofibers including vitamins indicated higher bioaccessibility in the food samples stored at 4 °C for 3 days.^23^

Fish is known to provide high levels of constituents such as fats and proteinous contents, which are important for the human diet. On the other hand, these constituents make fish highly perishable. Therefore, to inhibit microbial spoilage and oxidative deterioration, applications of food additives and/or irradiation have been applied.^24^ Scientists and food producers have sought innovative food technologies called a novel combination that can provide a ready-to-eat product treated with advanced technologies such as nanotechnology. Recently published studies based on nanoparticles, nanoemulsions, and nanoliposomes reveal this fact to the researchers.^25−27^ So far, different nanotype materials like citrus oils, thyme, curcumin, nisin, and rosemary fabricated with the electrospinning technique were effectively utilized to delay spoilage in fish meat. In addition, zein having good elasticity, film-forming capacity, and hydrophobicity properties is used for electrospinning applications to obtain the coating or packaging materials for foods.^28,29^ Moreover, cooking techniques like sous vide cooking have been already combined with novel applications to protect the quality of the salmon meat samples.^30^ However, to our knowledge, there has been no study in the literature on the combined effects of coating of Rainbow trout (*Oncorhynchus mykiss*) by zein nanofibers containing ground laurel leaves and its air-frying cooking on the quality parameters. The objectives were to fabricate and characterize LZn and then to investigate the combined effects of LZn coating and air fryer cooking of fresh fish fillets on their quality parameters. LZn-coated and air-cooked samples were stored at 4 °C, and their oxidative and microbiological stabilities, color, and sensory properties were monitored including control samples for 10 days.

## 2 Materials and Methods

### 2.1 Materials

Dried laurel leaves of *L. nobilis* L. were obtained from a local market in Istanbul, Turkiye. Zein from maize (Z3625) was purchased from Sigma-Aldrich (St. Louis, USA). Ethanol (96%, v/v) was provided by Merck (Darmstadt, Germany). Rainbow trout (*O. mykiss*) were obtained from a local aquaculture company in Van, Turkiye.

### 2.2 Methods

#### 2.2.1 Electrospinning Encapsulation of GLL

The zein solution was prepared at 15% (w/v) in ethanol solution (80%, v/v). Then, GLL (1 g) was added to the solution. The blend was stirred at 25 °C for 24 h to ensure homogeneity. For nanofiber fabrication, electrospinning equipment with a syringe pump (New Era Pump Systems Inc., NE-300, USA) and a voltage power supply (Nanofen, Ankara, Turkiye) was used. The feed rate, applied voltage, and distance to the collector plate during electrospinning were 0.8 mL/h, 17 kV, and 15 cm, respectively.

#### 2.2.2 Fish Samples, Nanocoating, and Cooking

The rainbow trout samples (*n* = 3; size of fish fillets: weight: 40 ± 1.748 g, thickness: 1.78 ± 0.63 cm, and length: 4.56 ± 0.58 cm) were skinned and debonned. All fish meat samples were stored at 4 ± 2 °C for 2 h after they were coated with LZn. Then, noncoated rainbow trout fillets (control samples) and coated samples with LZn were cooked using a cooking machine called an air fryer (Xiaomi Mi Smart Air Fryer, China) at 180 °C for 15 min at *n* = 3 for each analysis day (3, 6, 8, and 10 days) and each group was prepared separately. A total of 24 samples were stored in a cold freezer during the analysis period. After cooking, all samples were cooled to room temperature for 6 min. All cooled fish meat samples were stored within house-type storage boxes at 4 °C for 10 days.

#### 2.2.3 Scanning Electron Microscopy (SEM)

Scanning electron microscopy (Leitz, AMR 1000, Germany) was used for morphological analysis of the nanofibers. The average fiber diameters were obtained from software (Image).

#### 2.2.4 Fourier Transform Infrared Spectroscopy (FTIR) Analysis

The infrared spectra of samples were measured by using an FTIR spectrometer equipped with the ATR diamond module (Bruker Optics, Germany) in the range of 400 and 4000 cm^–1^ wavenumbers with a resolution of 4 cm^–1^.

#### 2.2.5 Total Mesophilic Aerobic Bacteria (TMAB)

TMAB growth in all fish meat samples was investigated according to the method reported by Maturin and Peeler.^31^ A fish sample (10 g) and peptone water (90 mL) were homogenized for 150 s by using a Stomacher (IU Instruments, Spain). The dilutions (to 10^5^) were prepared. All samples (*n* = 12) were incubated at 35 °C for 48 h. TMAB results were reported in terms of log colony forming units (CFU)/g for each sample.

#### 2.2.6 Thiobarbituric Acid (TBA) Analysis

The TBA analysis (*n* = 3) was conducted as stated by Tarladgis et al.^32^ Homogenized fish samples (control and LZn-coated) (10 g), distilled water (97.5 mL), and 4 N HCl (2.5 mL) were placed in the flasks and heated to collect the distillate. The distillate (5 mL) was taken out and placed in a glass tube. The tube was heated at 70 °C for 30 min. As soon as the color difference was obtained in the tube, the absorbance was recorded at 538 nm by using a spectrophotometer (UV-1900PC, AOELAB instrument, Shanghai, China). The obtained TBA results for each fish meat sample were reported as milligrams of malondialdehyde per kilogram (mg of MDA/kg) in fish meat.

#### 2.2.7 Color Stability

The model CR-400 Konika Minolta colorimeter (Japan) was used to measure L*, a*, and b* values for all samples (*n* = 3). Lightness, black to white between 0 and 100, is defined as L*, and the a* value refers from red (+) to green (−), while the b* value determines yellow (+) and blue (−) according to the CIELab system.^33^ In addition, the total color difference (Δ*E*) was obtained as follows: Δ*E* = [(L*_1_ – L*_2_)^2^ + (a*_1_ – a*_2_)^2^ + (b*_1_ – b*_2_)^2^]^1/2^.

#### 2.2.8 Sensory Evaluation

The sensory evaluation in the fish meat samples was determined using scores between 0 and 10 points. The values of <5 were determined to be unacceptable, while the odor, texture, taste, and color of all samples (control fish meat: without coating; fish meat: coated with LZn) were analyzed by 10 trained panelists. Random codes were used for the samples to the panelists. Samples on the white plates were given to the panelists in a room that was well lit and ventilated.^34^

#### 2.2.9 Statistical Analysis

The results obtained from control fish meat samples and the fish meat samples treated with LZn for 10 days under cold storage conditions (4 °C) were subjected using analysis of variance. JMP statistical discovery software (SAS) was used to define the significant differences between the two group samples. The LSD means were obtained from Student’s *t* test.

## 3 Results and Discussion

### 3.1 SEM Analysis

**Figure 1:**
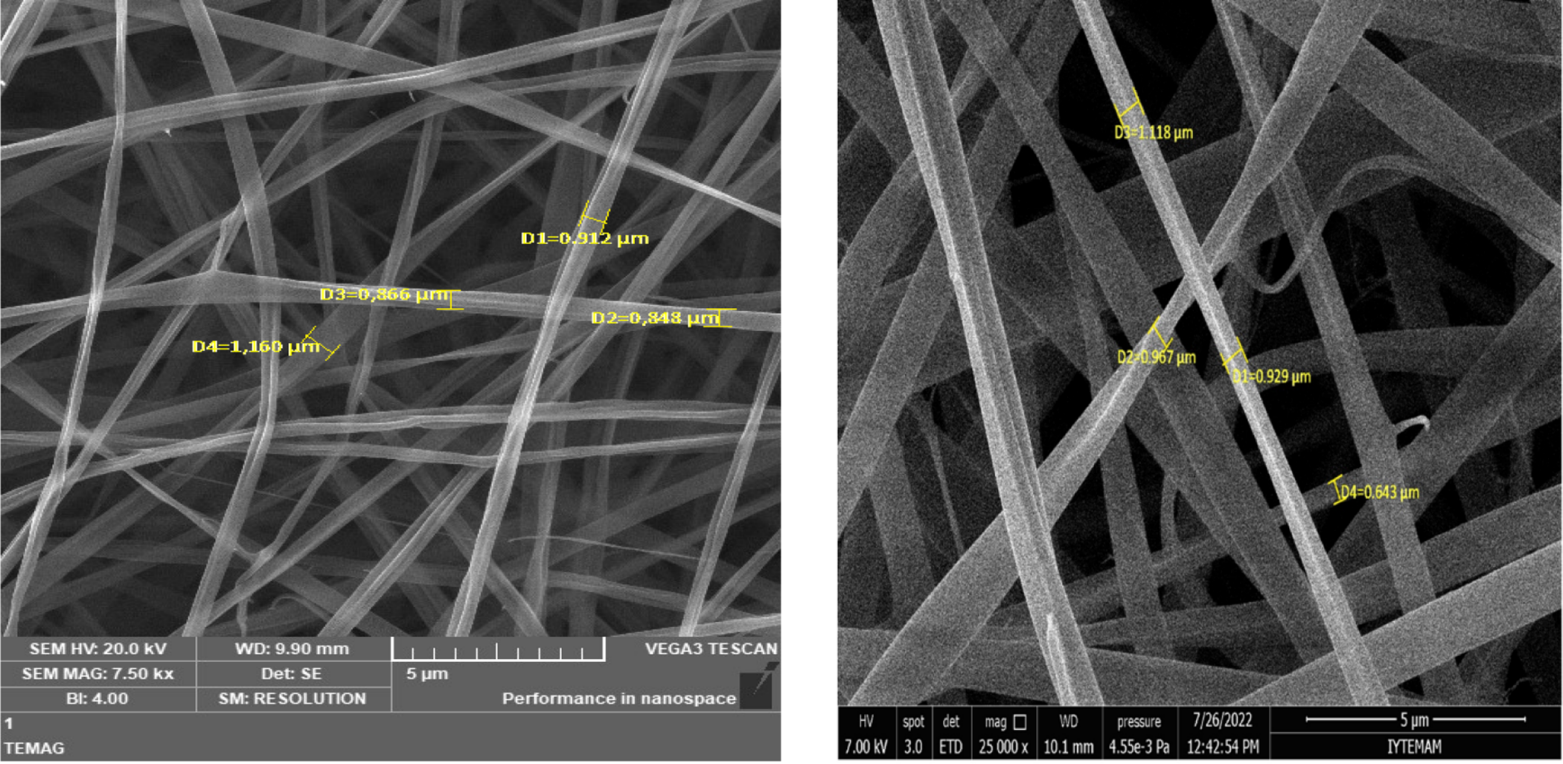
SEM images of zein nanofibers (A) (7500×) and LZn (B) (25,000×).

SEM images of Zn with and without GLL are displayed in Figure 1. The diameter of Zn was 307.5 ± 54.3 nm, while the incorporation of GLL to Zn made the diameter of the obtained fibers 335.8 ± 43.6 nm. As seen from the figures, uniform and beadless nanofibers were produced from zein with and without GLL. The SEM images showed that the fiber formation was not affected by the incorporation of GLL into the solutions. It has been reported that the incorporation of cinnamon essential oil into zein solution did not significantly change the diameters of nanofibers.^35^ In addition, the diameters in the ranges of 677 ± 26.9 and 697 ± 58.2 nm were white networks without any agglomeration. It has been stated that Zn with laurel essential oil gives a smoother surface without beads.^28^

### 3.2 FTIR analysis

**Figure 2:**
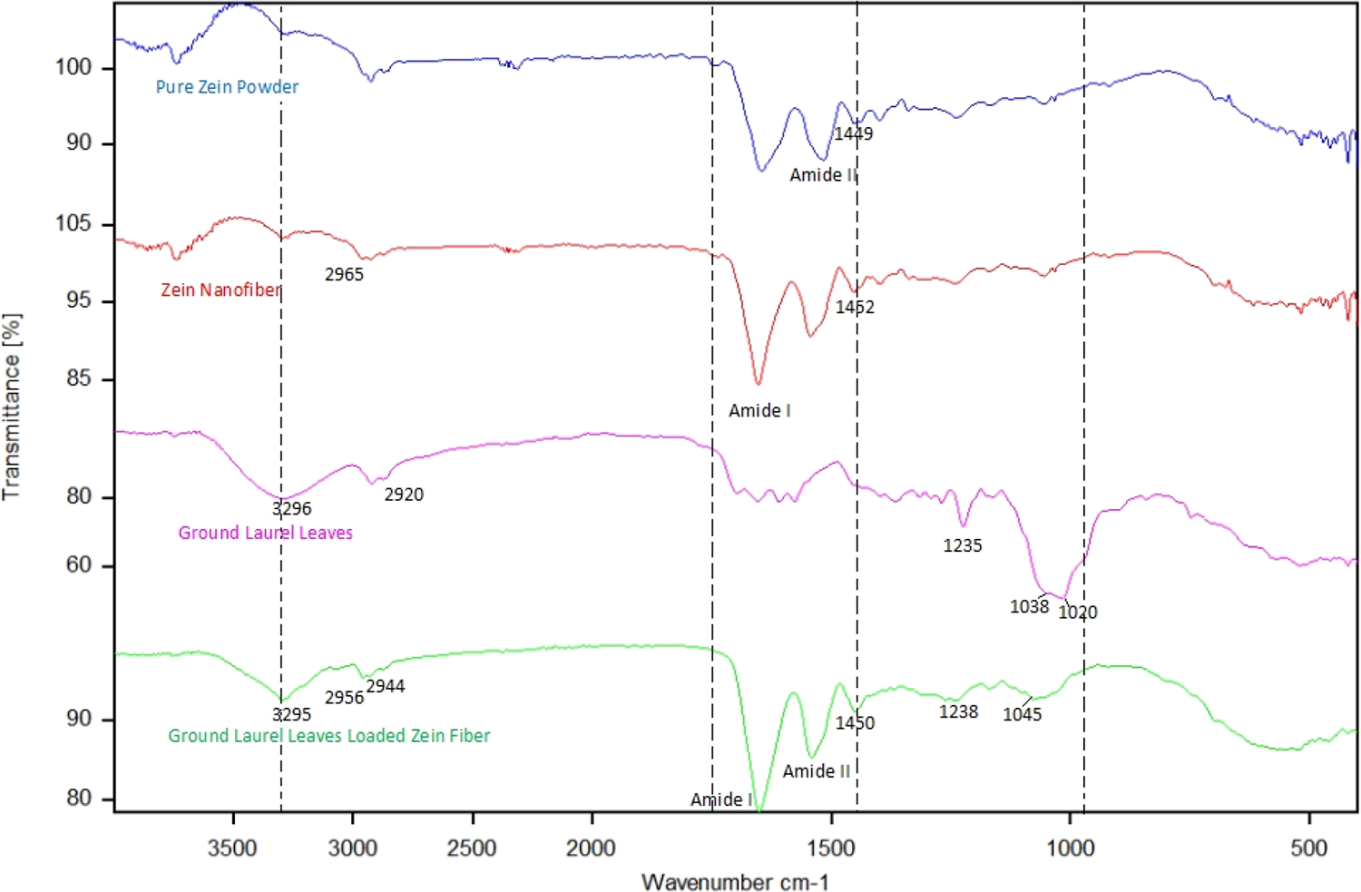
FTIR spectra of pure zein powder (A), zein nanofiber (B), GLL (C), and LZn (D).

Figure 2 shows the ATR-FTIR spectra of the zein powder, Zn, GLL, and LZn. The FTIR results of the zein powder exhibited absorbance bands of intensities for −OH and −NH groups at 3295 cm^–1^, C–N stretching at 1449 cm^–1^, amide I at 1651 cm^–1^, amide II at 1518 cm^–1^, amide III at 1239 cm^–1^, and carboxylic acids at 2927 cm^–1^ corresponding to the vibrations of inter-ring chain and aromatic rings (1610, 1580, 1360, 1299, and 779 cm^–1^).

The FTIR results of Zn display characteristic peaks at 3283 cm^–1^ (stretching vibrations of hydroxyl),^36^ 2944, 2956, and 2900 cm^–1^ (CH stretching vibrations of CH2 and CH3 aliphatic groups), and 1650, 1540, and 1260 cm^–1^, which correspond to C=O stretching vibrations, N–H bending vibrations, and C–N stretching band, respectively.^37,38^ The characteristic bands of GLL between 1700 and 1500 cm^–1^, 3000 and 2800 cm^–1^, and 3500 and 3300 cm^–1^ indicate the presence of C–O stretching of carboxyl groups, symmetrical and asymmetrical C–H stretch for alkane, and O–H stretch, respectively. Moreover, the absorption peaks observed at 1670–1600 and 900–700 cm^–1^ were related to alkene C=C stretch and aromatic C=C, respectively.^39^ These absorptions are important for recognizing GLL.

In the spectra of LZn, the bands of zein were observed at 1660, 1540, and 1238 cm^–1^ and GLL in regions at 3320, 1660, 1520, 1450, and 1235 cm^–1^, confirming the loading and encapsulation of GLL into the matrix. After electrospinning, the peaks at 2920 and 1640 cm^–1^ indicate the chemical interaction of GLL with the zein network. With GLL incorporation, the absorption peaks at 1760 and 1690 cm^–1^ in the GLL spectrum were shifted to a lower wavenumber (1680 cm^–1^) in LZn. The hydrophobic and electrostatic interactions between the aromatic groups of zein and the functional groups of GLL were clearly observed. Similar results for barije (*Ferula gummosa* Boiss) essential oil and eucalyptus encapsulated in zein nanofibers have also been reported.^28^

### 3.3 TMAB

TMAB growth in the control and other samples is presented in Table 1. The initial load of the raw fish meat samples that would be processed was found to be lower than 3.26 log CFU/g (*n* = 12). A rapid increase in TMAB growth for the control sample was observed as the storage time at 4 °C increased (*p* < 0.05). Despite the increase in the control sample hand, the TMAB increase was more effectively delayed for LZn-coated samples (*p* < 0.05). Moreover, the average limitation as a percentage compared to the control was determined to be 3.44, 45.21, and 40.06% on the third, sixth, and eighth days of cold storage, respectively (*p* < 0.05). On the eighth day, when the control samples reached 3.22 log CFU/g, the TMAB load of LZn-treated samples was found to be 1.93 (*p* < 0.05). As stated by the report^40^ (Guidelines for Assessing the Microbiological Safety of Ready-to-Eat Foods Placed on the Market) revealed by the Health Protection Agency in 2009, food cooked for sale or consumption at <10^3^ CFU/g was defined as satisfactory, but 10^3^ < 10^5^ CFU/g had been accepted as a borderline for these kinds of food. The present study’s TMAB results were also associated with sensory quality parameters. Therefore, nanocoating application successfully limited TMAB in cooked fish samples stored at 4 °C. It has been stated that the nanocoating process could effectively limit TMAB in raw food samples.^41^ In this respect, some studies based on nanoemulsion used for raw fish meat revealed that the TMAB load of fish fillets having different initial TMAB loads (between 3 and 4 log CFU/g) decreased to 2.15 log CFU/g.^42^ In addition to nanoemulsion used for raw fish fillets, it has been reported that chitosan nanoparticle application delayed the TMAB growth by 0.84 log CFU/g in fish finger samples.^43^ It has also been reported that nanofiber treatment on fish meat could limit TMAB up to 3 log CFU/g.^44^ In this study, the fish sample coated with LZn was left for 2 h before cooking to determine the effect of LZn. Even the 2 h effect of LZn (335.8 ± 43.6 nm average size) and then air fry cooking of rainbow trout fish fillets provided a good limitation in TMAB growth (*p* < 0.05). Excluding nanoapplications related to fish quality, different cooking techniques have already been used for fish fillets. For example, the rainbow trout fillets cooked with the sous vide method (vacuum-sealed in a cooking pouch: limited oxygen) had a 3.16 log CFU/g TMAB count at the end of 18 days.^45^ Enough cooking can kill the bacterium, but some potential preservation methods can be afforded because of the fact that there may be active toxins and high contamination risks. Therefore, this novel nanofiber application combined with air fryer cooking techniques might provide a guiding role in decreasing the potential contamination risk in cooked fish meat and increasing food safety for consumers.

**Table 1:** Some Quality Parameters of Control and LZn-Coated Rainbow Trout Fillets^a^

		**storage day**			
group	analysis	3rd day	6th day	8th day	10th day
control	TMAB	2.03 ± 0.05^aB^	3.76 ± 0.68^aA^	3.22 ± 0.23^aA^	ND
	TBA	2.90 ± 0.08^bA^	3.49 ± 0.57^aA^	3.13 ± 0.04^aA^	3.33 ± 0.08^aA^
	L*	29.69 ± 0.90^aA^	33.90 ± 0.28^aA^	10.72 ± 7.40^bC^	18.42 ± 1.73^aB^
	a*	10.84 ± 0.77^aA^	17.10 ± 0.22^aA^	–36.99 ± 13.54^aB^	6.83 ± 0.61^aA^
	b*	–0.31 ± 0.64^bB^	5.94 ± 1.68^aA^	–104.09 ± 20.88^bC^	10.06 ± 0.25^aA^
LZn-treated	TMAB	1.96 ± 0.05^aA^	2.06 ± 0.11^bA^	1.93 ± 0.11^bA^	ND
	TBA	3.51 ± 0.08^aB^	2.57 ± 0.03^aD^	3.27 ± 0.02^aC^	3.36 ± 0.12^aBC^
	L*	31.11 ± 1.81^aB^	34.25 ± 0.39^aA^	29.19 ± 0.94^aB^	18.67 ± 0.88^aC^
	a*	11.05 ± 1.14^aAB^	16.25 ± 1.23^aA^	5.72 ± 2.50^aB^	–25.07 ± 6.54^bC^
	b*	7.44 ± 1.92^aA^	11.07 ± 3.44^aA^	–23.98 ± 6.06^aB^	–82.89 ± 10.56^bC^
	Δ*E*	7.60	5.21	92.64	98.27

a Superscripts a and b: within each column, different superscript lowercase letters show differences between treatment groups for the same storage day (*p* < 0.05). Superscripts A–C: within each row, different superscript uppercase letters show differences between the storage days within the same analysis group (*p* < 0.05). ND: not detected.

### 3.4 Thiobarbituric Acid (TBA)

The oxidation in foods could be evaluated as one of the quality parameters; thus, the TBA analysis could be used to monitor lipid oxidation, especially for fatty foods.^27,46,47^ Either lower or higher TBA than the initial values for fish fillets could be attained during cold storage.^48^ In this study, the TBA value of the control on the third day of storage was found to be lower as compared to the cooked fish fillets with an air fryer after treatment of nanofibers (*p* < 0.05). The LZn application decreased the TBA value from 3.51 to 2.57 mg MDA/kg (*p* < 0.05). After the third day, the b* value associated with the oxidation of the samples with ZLn also started to be unstable. On the other hand, after the sixth day, the TBA value reached 3.36 mg MDA/kg, but no significant differences were observed between the two groups (*p* > 0.05). As could be seen in recently published study results, salmon and chicken meat samples treated with nanofibers obtained from sesame oil provided higher oxidative stability as compared to the untreated samples during the analysis period.^49^ Furthermore, beef samples coated with nanofibers fabricated with eugenol-loaded gelatin had a positive effect on color stability (b* value) associated with the acceleration in TBA value.^50^ These studies also supported the use of nanofibers on cooked or raw food materials to limit rapid oxidation. Also, it is widely known that wrong cooking may also cause rapid oxidation in foods, but the air fryer cooking technique did not increase the oxidation level in fish meat samples.

### 3.5 Color Stability

The L*, a*, b*, and Δ*E* results of samples are given in Table 1. The Δ*E* value on the third day of storage was defined as 7.56, but that of the samples on the sixth day was found to be 5.21. The Δ*E* value is defined depending on different parameters like cooking type, processing conditions, types, protein structures, degradation level in the pigment, storage period, and treatments as reported by Teixeira et al.^51^ and Bedane et al.^52^ Furthermore, it has been reported that industry could be utilized to determine the meat color changes as “acceptable” (7 < Δ*E* < 9) and also “unacceptable” (Δ*E* ≥ 9) qualities.^53^ With the present study, excluding the Δ*E* values on the third and sixth days at 4 °C, on the next days, it was observed that Δ*E* values extensively exceeded the above-mentioned scales. In addition to the Δ*E*, b* values associated with potential oxidation^54^ of C samples were highly unstable (change: −0.31 to −104.09 on the eighth day), while b* values (change: 7.44 to −23.98 on the eighth day) in LZn-coated fish samples were more stable compared to the control. In the present study, Δ*E* and b* values provided meaningful differences, but L* and a* values did not show a remarkable difference. In terms of color quality, especially the Δ*E* showed the important differences between the control and the sample coated with LZn at the eighth day of storage.

### 3.6 Sensory Evaluation

The odor, color, texture, and overall acceptability (OVa) of control samples and the samples coated with LZn are presented in Table 2. Three days later, following the initial processes, odor, texture, and also OVa (8.66, *p* < 0.05) were higher as compared to the control. It has been reported that nanomats containing nisin and curcumin were able to delay the sensory deterioration in fish samples stored at 4 °C.^44^ It has also been stated that sensory acceptability in the salad dressings with extracts from humid laurel leaves was defined as higher as compared to the control stored at 10 °C.^55^ In addition, the leaves are defined as characteristically fragrant once crushed yet taste bitter and aromatic as stated by Bagchi and Srivastava.^56^ In the present study, especially the odor parameter in the cooked fish samples coated with LZn was significantly high (*p* < 0.05). Nanomaterials have a very high contact area to interact with the surface of the food material due to their nanoscale dimensions. In this study, especially during 6 days of cold storage at 4 °C, a better sensory property (OVa: 5.66; *p* < 0.05) was observed as compared to the control. Microbiological spoilage in meat products especially is correlated with the deterioration in odor quality. While the TMAB count was found to be lower in the group treated with nanomaterials with an air fryer cooking process, odor acceptability in the same samples was also found to be higher during 8 days.

**Table 2:** Sensory Evaluation of Control and LZn-Coated Rainbow Trout Fillets^a^

		**storage day**			
group	analysis	3rd day	6th day	8th day	10th day
control	odor	7.33 ± 0.52^bA^	4.50 ± 0.54^bB^	3.83 ± 0.40^bC^	3.33 ± 0.51^bC^
	color	8.50 ± 0.83^aA^	5.33 ± 0.51^aB^	4.00 ± 0.00^aC^	3.33 ± 0.51^bC^
	texture	7.83 ± 0.40^bA^	6.00 ± 0.89^aB^	4.33 ± 0.51^bC^	3.83 ± 0.98^aC^
	overall acceptability	7.83 ± 0.40^bA^	4.83 ± 0.40^bB^	4.00 ± 0.00^bC^	3.33 ± 0.51^bD^
LZn-treated	odor	8.66 ± 0.52^aA^	5.00 ± 0.00^aB^	4.66 ± 0.40^aBC^	4.16 ± 0.40^aC^
	color	8.00 ± 0.89^aA^	6.00 ± 1.20^aB^	4.33 ± 0.81^aC^	4.50 ± 0.54^aC^
	texture	8.66 ± 0.51^aA^	5.83 ± 1.32^aB^	5.00 ± 0.00^aBC^	4.33 ± 0.51^aC^
	overall acceptability	8.66 ± 0.51^aA^	5.66 ± 0.51^aB^	4.50 ± 0.54^aC^	4.66 ± 0.51^aC^

a Superscript a and b: within each column, different superscript lowercase letters show differences between treatment groups for the same storage day (*p* < 0.05). Superscript A–C: within each row, different superscript uppercase letters show differences between the storage days within the same analysis group (*p* < 0.05). ND: not detected.

## 4 Conclusions

The electrospun solution containing GLL and zein nanofibers (335.8 ± 43.6 nm) was effectively fabricated into LZn, and then this fabrication was successfully proved with the characterization analysis. TMAB growth in the fish coated with LZn and cooked with an air fryer cooking technique was limited, while this combination provided a higher sensory acceptance compared to the control. Rapid oxidation in the fish samples with LZn and then cooking with the air fryer cooking technique that needs the usage of a little oil was limited in the initial period of the cold storage period. Furthermore, Δ*E* results showing the color differences between the two products clearly reflected the quality changes after the sixth day. The microbial limitation as a percentage reached 40% (1.93 to 3.22 log CFU/g), and overall acceptability was increased (3.33 to 4.66) by 39% (*p* < 0.05). The study related to air fryer cooking and LZn treatment declared promising results for the next step in food industry applications.
